# Adult Ileocolic Intussusception Secondary to Cecal Lipoma: A Case Report

**DOI:** 10.7759/cureus.59986

**Published:** 2024-05-09

**Authors:** George Angelakakis, Sarah Fish, Kenneth D Katz

**Affiliations:** 1 Department of Emergency and Hospital Medicine, Lehigh Valley Health Network/University of South Florida Morsani College of Medicine, Bethlehem, USA; 2 Department of Emergency and Hospital Medicine, Division of Medical Toxicology, Lehigh Valley Health Network/University of South Florida Morsani College of Medicine, Allentown, USA

**Keywords:** gastroenterology, hemicolectomy, colon, intussusception, lipoma

## Abstract

Large intestinal intussusception is rare in adults. Among potential pathologic lead points for intussusception are lipomas, benign tumors very infrequently found in the large bowel. A 30-year-old woman presented to the emergency department with a chief complaint of generalized abdominal pain for two weeks. A computed tomography scan of her abdomen and pelvis showed an ileocolic intussusception with a lead point of 6.7 cm. The lead point appeared to be predominantly fat. A colonoscopy revealed a large, obstructing lesion in the transverse colon. The patient underwent exploratory laparotomy with a right hemicolectomy, and a pathologic diagnosis of a lipoma was made. The patient recovered from surgery without complications and returned to her normal diet three weeks after discharge. This case highlights an unusual and rare presentation of an ileocolic intussusception caused by a cecal lipoma acting as a lead point.

## Introduction

An intussusception occurs when a proximal portion of the intestine invaginates into a distal portion of the intestine telescopically [1]. Intussusception in adults is rare, representing only 1% of patients with bowel obstructions and 0.003%-0.02% of global hospital admissions [2]. Intussusception is much more common in pediatric patients, accounting for 95% of cases [3]. In addition, while intussusception is usually idiopathic in children, in adults, 90% of cases arise due to secondary causes that serve as lead points, such as benign and malignant neoplasms, polyps, and colonic diverticula [4]. Among benign neoplasms, lipomas rank third after hyperplastic and adenomatous polyps. A review of the literature reveals one in five lipomas in the large intestine present in the cecum, with lipomas acting as lead points in 17% of all intussusception cases [5]. Lipomas occur very rarely in young adults; instead, they tend to occur later in life [1,5]. In this report, we present the case of a 30-year-old woman with ileocolic intussusception caused by an underlying cecal lipoma acting as a lead point and treated by a right hemicolectomy via exploratory laparotomy. Parts of this case have been submitted for presentation at the 2024 Pennsylvania American College of Emergency Physicians Scientific Assembly on May 3, 2024.

## Case presentation

A 30-year-old woman presented to the emergency department (ED) with two weeks of generalized abdominal pain. Her relevant past surgical history included a gastrostomy tube in childhood secondary to a traumatic vehicle injury. She had previously been evaluated for this complaint at an urgent care one week before the ED presentation and had an unremarkable abdominal X-ray. She again visited urgent care several days later and was then advised to go to the ED due to unremitting abdominal pain and a heart rate exceeding 160 beats per minute.

**Figure 1:**
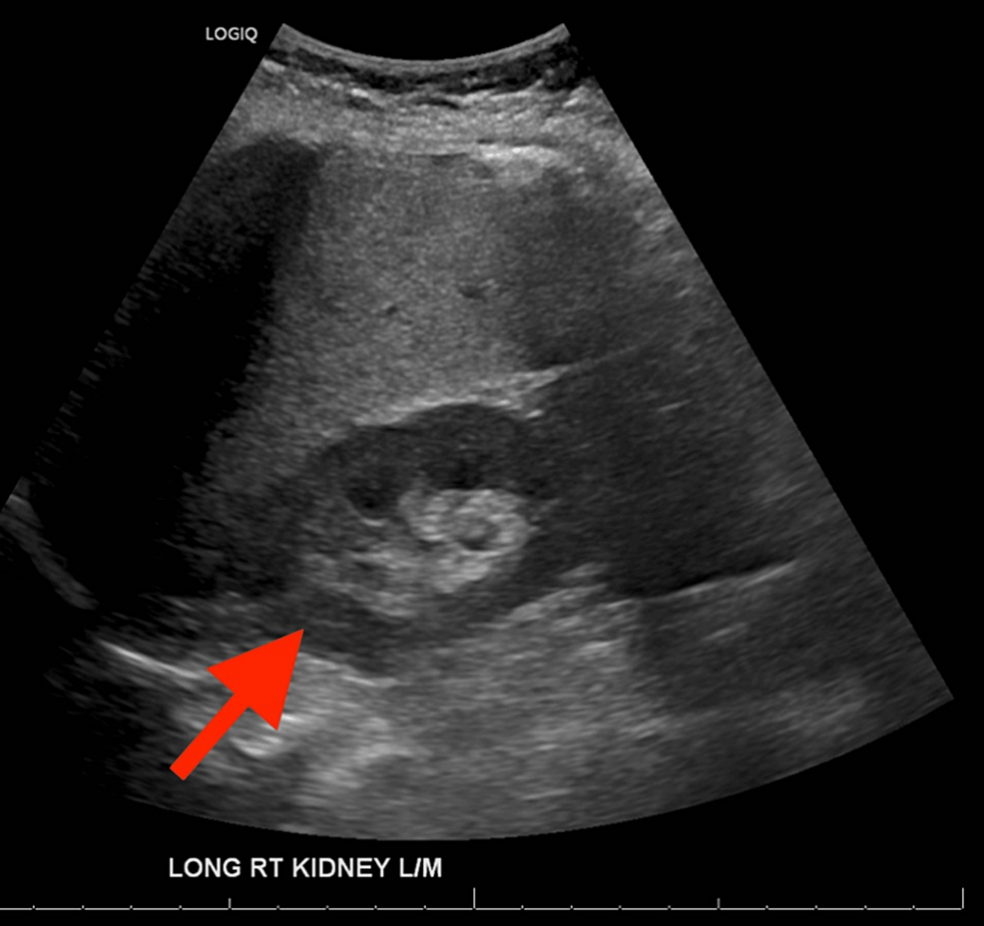
Right upper quadrant ultrasound demonstrating hydronephrosis (red arrow)

On ED arrival, the patient rated her pain as 6 out of 10 and described it as a pressure and stabbing sensation generalized throughout her entire abdomen without vomiting, hematochezia, or fever. Vital signs included blood pressure 130/100 mm/Hg, heart rate 103 beats per minute, respiratory rate 18 breaths per minute, temperature 99.9°F (37.7°C), and SpO_2_ 97% on room air. Physical examination was significant for a soft, mildly distended abdomen with normal bowel sounds and generalized abdominal tenderness. An ultrasound of the right upper quadrant showed hepatic steatosis and mild right hydronephrosis (Figure 1).

**Figure 2:**
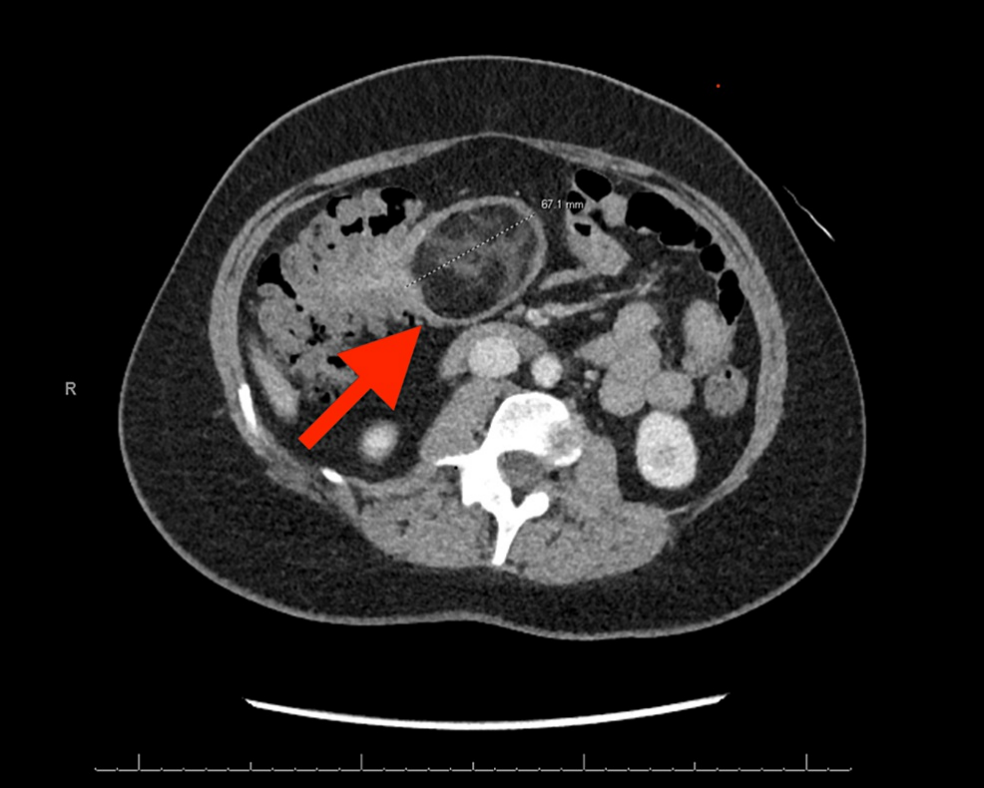
Contrast-enhancing CT axial view demonstrating lipoma as the lead point for ileocolic intussusception (red arrow) CT: computed tomography

**Figure 3:**
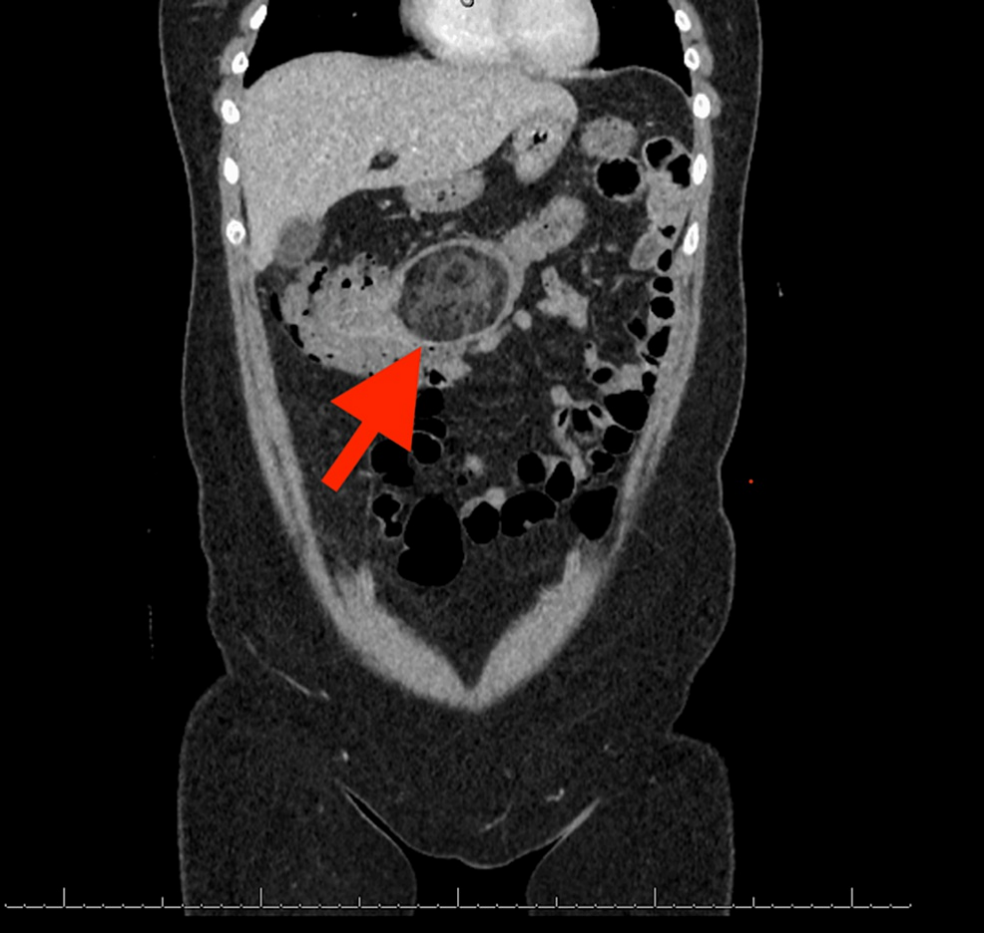
Contrast-enhancing CT coronal view demonstrating lipoma as the lead point for ileocolic intussusception (red arrow) CT: computed tomography

A computed tomography (CT) scan with intravenous contrast of the abdomen and pelvis was ordered due to suspicion of a more significant pathologic etiology, given her persistent complaints and nondiagnostic ultrasound findings. The CT demonstrated an ileocolic intussusception with a lead point of 6.7 cm (Figures 2, 3). On the CT scan, the lead point appeared as a predominantly fat attenuation lesion suggestive of a low-grade sarcoma; no free air was evident. The general surgery team was consulted, and the patient was admitted to the hospital.

**Figure 4:**
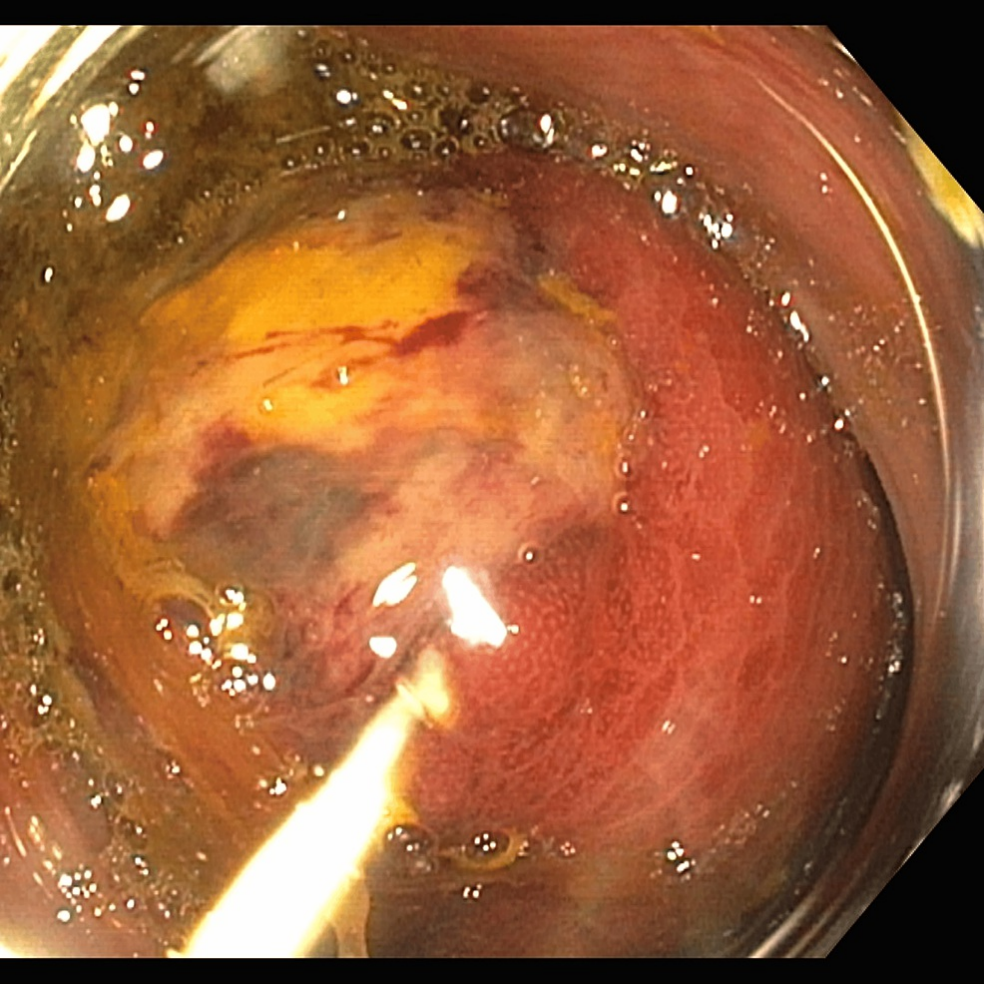
A colonoscopic image of the submucosal lipoma. This lesion led to partial bowel obstruction and an ileocolic intussusception

**Figure 5:**
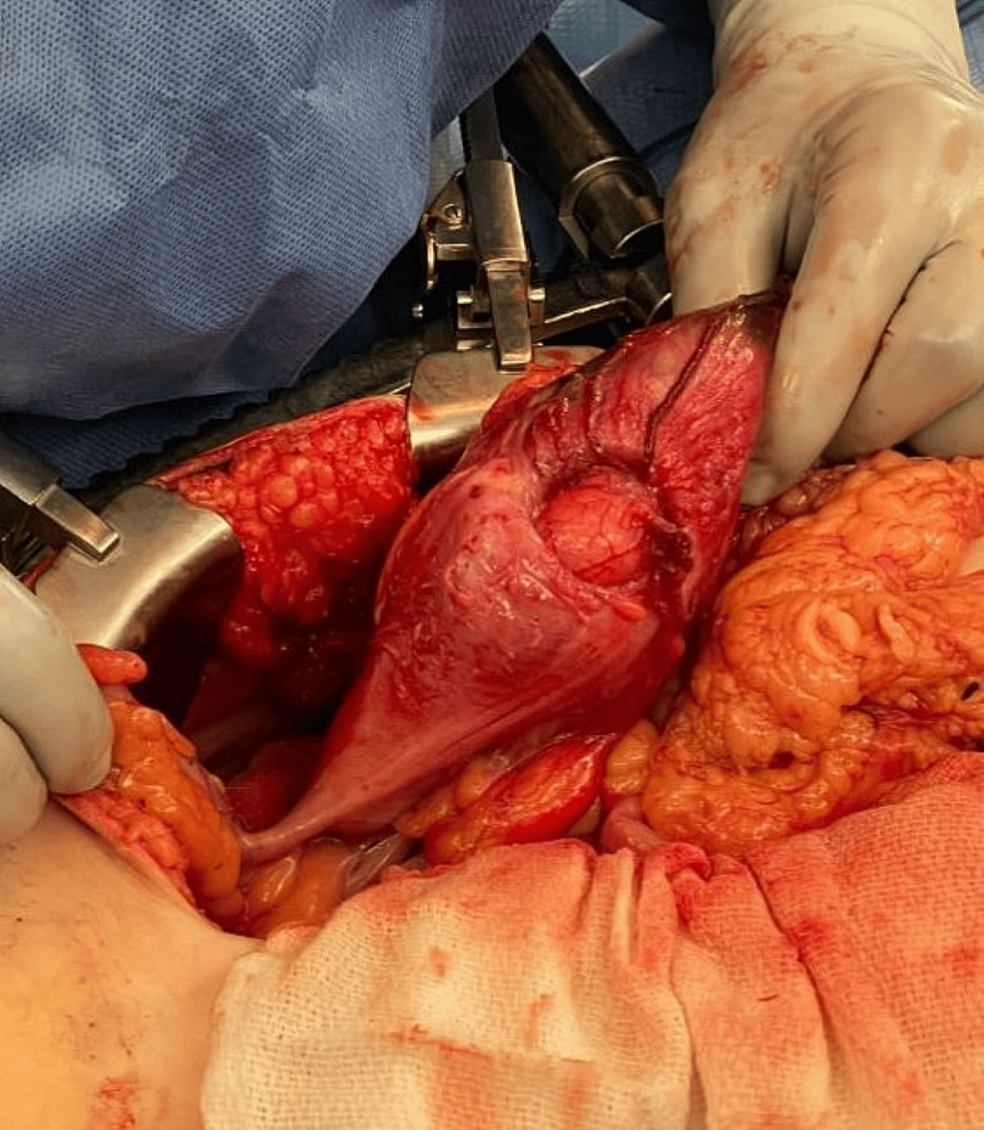
A visualization of the ileocecal lipoma within the surgical field

**Figure 6:**
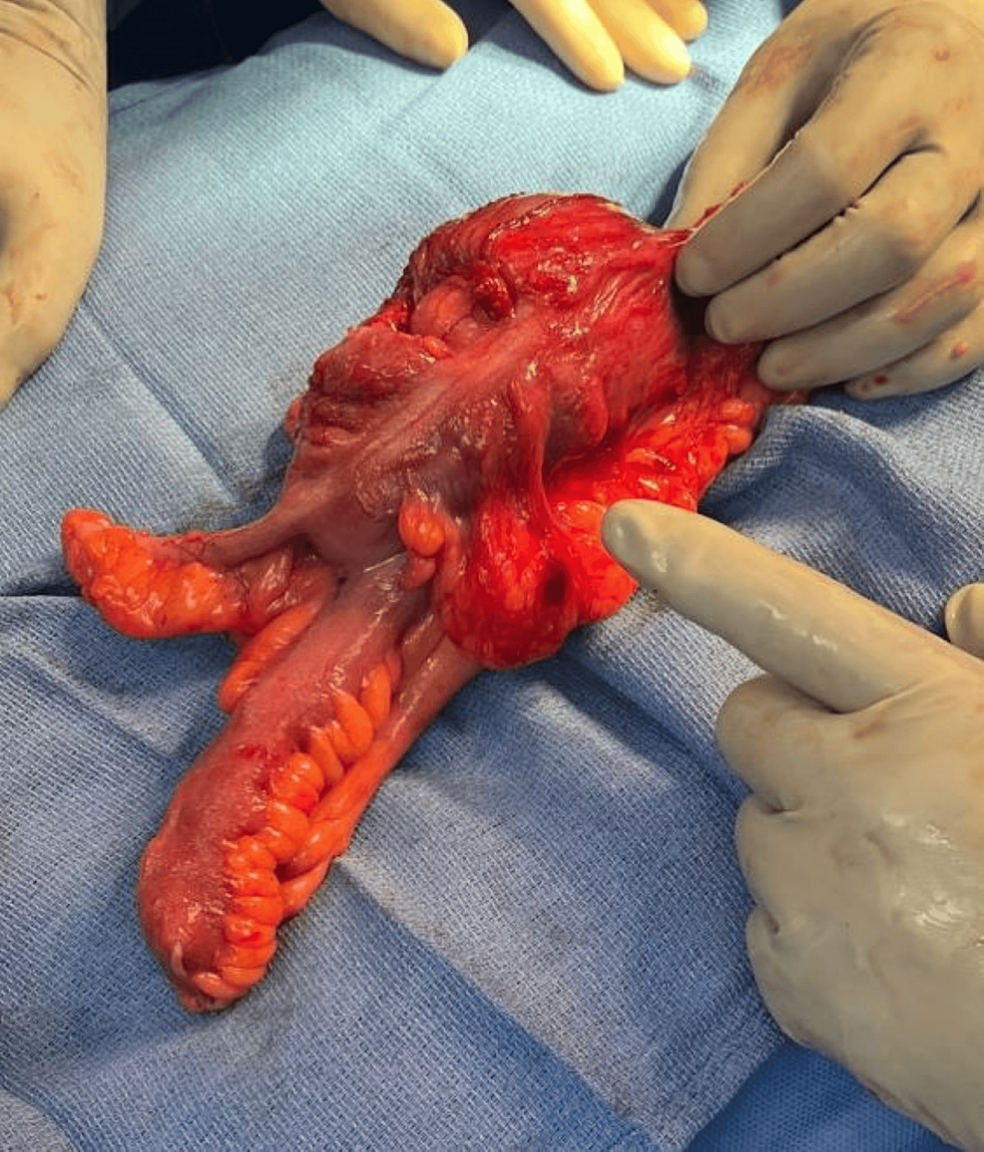
Postoperative image of the resected lipoma, showing part of ileum, cecum, and appendix

On hospital day (HD) 1, the gastroenterology team was also consulted, and a colonoscopy was performed on HD 2. The colonoscopy revealed a large, partially obstructing, ulcerated, and nonbleeding submucosal lesion in the transverse colon approximately 60-65 cm from the rectum, impeding the colonoscope from passing the region of concern (Figure 4). On HD 3, the patient underwent an exploratory laparotomy with a complication-free right hemicolectomy (Figures 5, 6). The subsequent pathology report identified the mass as a submucosal cecal lipoma. The patient was discharged on HD 9. During her follow-up appointments one and three weeks after being discharged, the patient noted a marked improvement in pain and tolerance to her usual diet.

## Discussion

Intussusception is classified into four categories according to its location along the bowel: enteroenteric involves only the small bowel, colocolic is exclusive to the large intestine, ileocolic is a prolapse of the terminal ileum into the ascending colon, and ileocecal occurs when the ileocecal valve serves as a lead point [6,7]. The typical presenting signs and symptoms for each of the four types of intussusceptions are nonspecific, including intermittent abdominal pain, nausea, vomiting, and diarrhea [8]. These obstructive symptoms are different from the typical abdominal pain, currant jelly stools, and a palpable tender mass common among children with intussusception [9]. Overall, intussusception is responsible for between 1% and 5% of bowel obstructions in adults [10].

After hyperplastic and adenomatous polyps, lipomas are the third most common benign tumor in the large intestine, with an incidence of 4.4% [11,12]. Within the large intestine, 19% of colonic lipomas are in the cecum, though the incidence of adult lipomas arising within the cecum of the large intestine is 0.836% [10,13]. Lipomas in the colon occur most commonly in women in their fifth and sixth decades of life, with a male-to-female incidence ratio of 1:5 [1,5]. Nearly 90% of colonic lipomas arise from the submucosa [14]. The patient described in this case was much younger than the typical patient suffering from a colonic lipoma.

Abdomen and pelvis CT is considered the imaging gold standard in diagnosing adult intussusception [2,15] and can distinguish between intussusception caused by and without a lead point. Abdominal X-rays, ultrasound, and upper gastrointestinal contrast/barium enema series may be utilized before CT but have lower diagnostic accuracy. A colonoscopy can be performed to visually confirm the diagnosis [6].

In contrast to children in whom barium or air enemas can be implemented as a therapeutic option, surgery is almost always used to treat adult intussusception. Surgery is also ultimately required for definitive diagnosis and treatment [7,15,16]. Other diagnostic and treatment options such as biopsy, radiation therapy, and chemotherapy are not typically explored to decrease the risk of possible seeding of tumor cells and bowel perforation [6,17].

## Conclusions

Adult cecal intussusception from a cecal lipoma acting as a lead point is a very rare condition. For patients presenting with typical obstructive bowel signs and symptoms (i.e., colicky abdominal pain, abdominal distension, nausea, and vomiting of unknown etiology), the most useful initial diagnostic imaging modality is CT of the abdomen/pelvis, which, in this case, identified a fatty mass. Subsequent colonoscopy was attempted to both diagnose and potentially treat the condition but could not be completed. Additionally, resection of the cecal lipoma via open surgery is the most effective treatment and is also the best method to definitively diagnose through biopsy. Thus, lipomas should remain among the differential diagnoses responsible for adult large bowel obstruction.
